# Serplulimab combined with gemcitabine, nab-paclitaxel, and stereotactic body radiotherapy versus gemcitabine and nab-paclitaxel as first-line treatment for recurrent or metastatic pancreatic ductal adenocarcinoma: a randomized, open-label, multicenter, phase III clinical trial (WGOG-PAN 006/ICSBR-2)

**DOI:** 10.3389/fimmu.2026.1817221

**Published:** 2026-05-13

**Authors:** Xin Wang, Pei Zhang, Dan Cao, Huanji Xu

**Affiliations:** 1 Division of Abdominal Tumor Multimodality Treatment, Cancer Center, West China Hospital, Sichuan University, Chengdu, Sichuan, China; 2 Division of Abdominal Tumor, Department of Medical Oncology, Cancer Center, Laboratory of Abdominal Tumor Immunology and Microenvironment, State Key Laboratory of Biotherapy, West China Hospital, Sichuan University, Chengdu, Sichuan, China

**Keywords:** gemcitabine, nab-paclitaxel, PDAC, SBRT, serplulimab

## Abstract

**Background:**

Pancreatic ductal adenocarcinoma remains a malignancy with a dismal prognosis, characterized by a median overall survival of less than one year in the metastatic setting despite standard-of-care chemotherapy regimens like gemcitabine plus nab-paclitaxel. The PDAC tumor microenvironment is notoriously immunosuppressive, rendering single-agent immune checkpoint inhibitors largely ineffective. Stereotactic Body Radiotherapy has emerged as a potential strategy to induce immunogenic cell death and remodel the TME. Based on promising Phase II data demonstrating a 78.48% 6-month progression-free survival rate with the triplet combination of GnP, Serplulimab and SBRT, this Phase III trial aims to validate the efficacy of this “radio-immuno-chemotherapy” strategy.

**Methods:**

This prospective, randomized, open-label, multicenter Phase III study will enroll 198 patients with recurrent or metastatic PDAC who are naive to systemic therapy for advanced disease. Participants will be randomized (1:1) to the Experimental Group receiving Serplulimab (300 mg IV, Q3W) combined with Gemcitabine 1000 mg/m² + nab-Paclitaxel 125 mg/m², Days 1, 8, Q3W and SBRT (33–50 Gy/5 fractions) initiated in Cycle 2, or the Control Group receiving GnP alone.

**Outcomes:**

The primary endpoint is Overall Survival. Secondary endpoints include progression-free survival, Objective Response Rate, Disease Control Rate, and safety profiles assessed via NCI-CTCAE v5.0. Exploratory endpoints include cyclic multiplex tissue staining assays to evaluate immune spatial interactions.

**Discussion:**

This study addresses the critical unmet need in advanced PDAC by evaluating a mechanistic synergy between cytotoxic debulking, radiation-induced immune priming, and checkpoint blockade. If successful, this regimen could establish a new standard of care.

## 1 Introduction

Pancreatic ductal adenocarcinoma (PDAC) is highly malignant with a high disease-related mortality rate. Over half of patients present at the metastatic stage, and these metastatic PDAC patients have an extremely poor prognosis, with an estimated 5-year survival rate of only about 3% (1–3). Over the past decade, chemotherapy has made significant advances in treating metastatic PDAC. Regimens such as GnP, FOLFIRINOX, and NALIRIFOX are now established as standard first-line treatments for metastatic PDAC (1, 4, 5). The GnP regimen has demonstrated superior tolerability in both clinical trials and real-world studies. Furthermore, the MPACT study enrolled a higher proportion of patients with an ECOG performance status ≥1 compared to the ACCORD 11 and NAPOLI-3 trials (1, 4, 5). However, overall, chemotherapy alone yields limited objective response rates in metastatic PDAC and is prone to resistance development. Median survival remains below 12 months, necessitating exploration of novel therapeutic approaches to improve treatment outcomes.

In recent years, immunotherapy targeting programmed cell death protein 1 (PD-1) and programmed cell death ligand 1 (PD-L1) has achieved significant breakthroughs in treating various advanced solid tumors. However, its application in PDAC has yielded suboptimal efficacy (6–8). This is attributed to the immunosuppressive tumor microenvironment (TME) formed by interactions among cancer cells, immune cells, cancer-associated fibroblasts (CAFs), and the extracellular matrix within PDAC tissues (9, 10). Consequently, PDAC is considered an immunologically “cold tumor,” where immunotherapy yields poor outcomes (11). Given the extremely low efficacy of PD-1 monotherapy in common MSS/pMMR PDACs, and the fact that MSI-H/dMMR cases constitute only approximately 1%-2% of the population, immunotherapy alone is unlikely to meet the needs of most patients (12, 13). Our prior research indicates that while the combination of GnP and toripalimab is safe, its overall efficacy remains limited. Benefits are primarily confined to a small subset of patients with specific immune phenotypes. Therefore, there is an urgent need to incorporate radiotherapy to expand the beneficiary population through local tumor reduction and enhanced immunogenicity (6). Stereotactic Body Radiotherapy (SBRT), as a hypofractionated radiotherapy modality, not only delivers a higher biological effective dose but also induces immunogenic cell death (ICD) and promotes tumor cell antigen presentation. This leads to an increase in tumor-reactive T cells, thereby enhancing synergy with immunotherapy (14). Therefore, exploring the combination of immunotherapy with GnP and SBRT may offer a viable approach to further improve the treatment landscape for recurrent or metastatic PDAC.

Clinical studies have demonstrated that SBRT combined with immunotherapy exhibits certain efficacy in the late-line treatment of PDAC (15). Furthermore, the Phase II Extend study demonstrated that SBRT or stereotactic ablative radiotherapy (SABR) combined with systemic chemotherapy significantly improved PFS in patients with oligometastatic PDAC compared to systemic chemotherapy alone (10.3 months vs. 2.5 months). with systemic immune activation observed in patients’ peripheral blood post-radiotherapy. This has prompted exploration of adding SBRT to chemotherapy plus immunotherapy to further improve patient outcomes (16). Previously, our team have initiated a single-arm Phase II clinical study evaluating GnP chemotherapy plus Serplulimab, a novel anti-PD-1 monoclonal antibody combined with SBRT as first-line therapy. Preliminary data from 47 completed patients demonstrated a 6-month PFS rate as high as 78.48%, with the combination therapy exhibiting favorable safety (17). Therefiore, we propose to conduct a randomized controlled phase III clinical trial to compare whether the GnP regimen (with SBRT combined with Serplulimab) versus the standard first-line GnP regimen can provide survival benefit in treatment-naive patients with recurrent or metastatic PDAC.

## 2 Methods and analysis

This study has been approved by the Biomedical Ethics Committee of West China Hospital, Sichuan University. The trial has been registered with the National Clinical Trials Registry (Registration Number: NCT07336953). The study will be conducted at West China Hospital of Sichuan University, Chengdu Shangjin Nanfu Hospital, and Chengdu Seventh People’s Hospital. All participants are required to sign written informed consent forms. Both the trial protocol and manuscript preparation adhere to the Standard Protocol Intervention Trial (SPIRIT) guidelines.

### 2.1 Study objectives

The primary endpoint is to evaluate the difference in overall survival (OS) between the GnP regimen plus SBRT combined with Serplulimab versus the GnP regimen as first-line therapy for patients with recurrent or metastatic PDAC.

For secondary endpoints, the study rigorously monitored progression-free survival (PFS), objective response rate (ORR), and disease control rate (DCR) through dual assessment by an Independent Radiological Review Committee (IRRC) and investigators. Specifically, the study refined the specific response rates for both irradiated and non-irradiated lesions. Additionally, the study will comprehensively evaluate the efficacy and tolerability of this combination regimen by incorporating time to progression (TTP), duration of response (DOR), and safety assessments based on the Common Terminology Criteria for Adverse Events (CTCAE) version 5.0.

Exploratory studies focus on elucidating mechanisms of therapeutic efficacy at the molecular and immune levels. These investigations employ next-generation sequencing (NGS), immunohistochemistry (IHC), and multicolor immunofluorescence (CmTSA) to conduct in-depth analyses of tumor tissue mutations and immune microenvironment characteristics (e.g., PD-L1, MMR). Concurrently, serial blood samples are collected to monitor changes in T cell subsets, inflammatory cytokines, and circulating tumor DNA (ctDNA).

### 2.2 Study design

**Figure 1:**
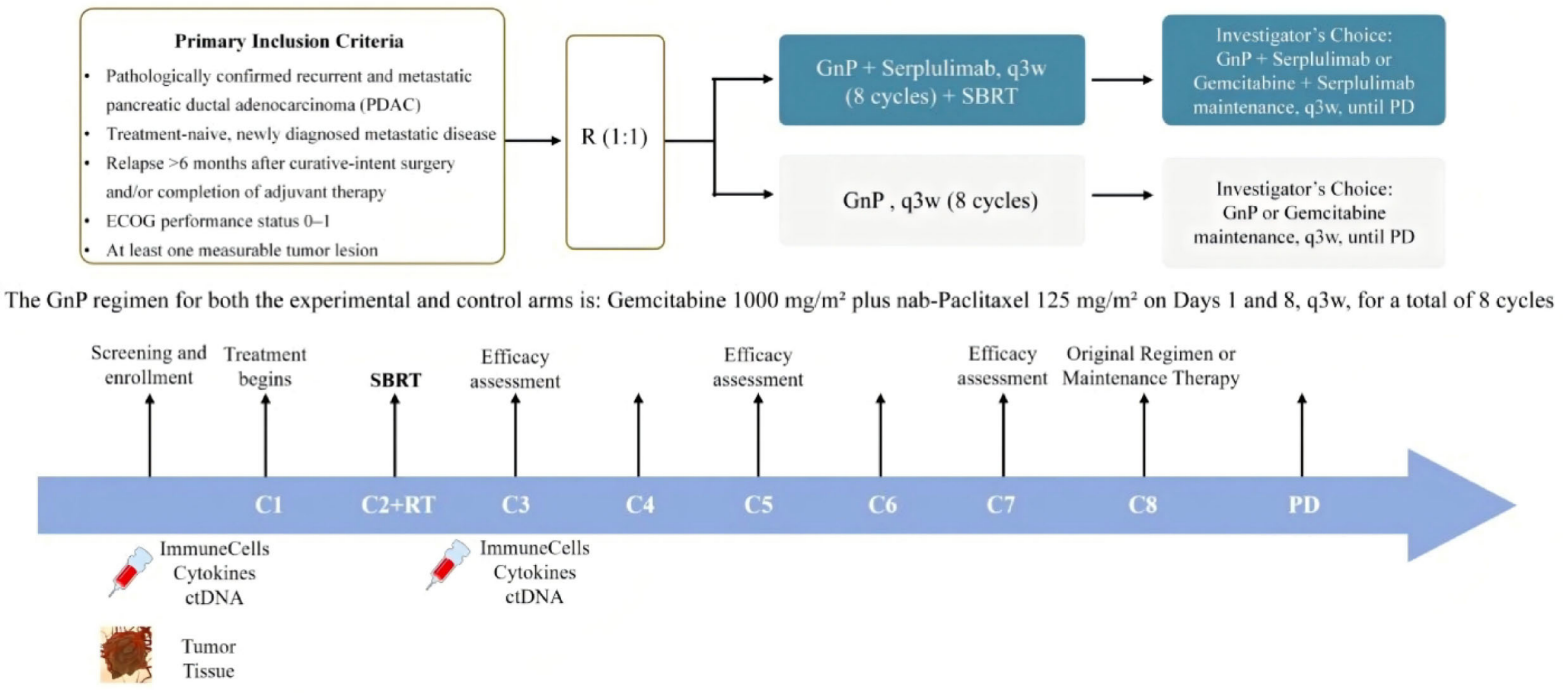
Research treatment process. This diagram illustrates the study design for treatment-naive patients with pathologically confirmed recurrent and metastatic PDAC. Eligible patients with an ECOG performance status of 0–1 and at least one measurable tumor lesion are randomized (1:1 ratio) into two study arms. In the Experimental Arm, patients receive the GnP regimen combined with Serplulimab q3w for 8 cycles followed by SBRT. In the Control Arm, patients receive the GnP regimen alone q3w for 8 cycles. The standard GnP regimen for both arms consists of gemcitabine (1000 mg/m²) and nab-paclitaxel (125 mg/m²) on Days 1 and 8, q3w. Following 8 cycles, patients proceed to maintenance therapy or the original regimen at the investigator’s choice until PD. Efficacy assessments occur at Cycles 3, 5, and 7. Exploratory biomarkers (immune cells, cytokines, and ctDNA) are collected at baseline, Cycle 3, and PD, with tumor tissue required at screening. C, cycle; ctDNA, circulating tumor DNA; D, day; ECOG, Eastern Cooperative Oncology Group; GnP, gemcitabine and nab-paclitaxel; PD, progressive disease; PDAC, pancreatic ductal adenocarcinoma; q3w, every 3 weeks; R, randomization; RT, radiotherapy; SBRT, stereotactic body radiation therapy.

This study is a randomized, multicenter, Phase III, open-label clinical trial for patients with recurrent or metastatic PDAC receiving first-line therapy (**Figure 1**). Subjects were assigned in a 1:1 ratio via an IWRS/IVRS system:

- Experimental Group (Group A): GnP regimen + Serplulimab + SBRT
- Control Group (Group B): GnP chemotherapy regimen

The GnP regimen in both the experimental and control groups is as follows: Nab-paclitaxel 125 mg/m², IV infusion, days 1 and 8, repeated every 21 days; Gemcitabine 1000 mg/m², IV infusion, days 1 and 8, repeated every 21 days. The immunotherapy and radiotherapy regimen in the experimental group is as follows: Serplulimab 300 mg, IV infusion, Day 1, repeated every 21 days, administered concurrently with Day 1 treatment of the GnP regimen during chemotherapy.

SBRT intervention begins in the second cycle, with a total of no more than five lesions receiving radiotherapy. The principles for selecting radiotherapy target lesions are as follows: no more than five (1–5) metastatic lesions will be selected for irradiation. For patients who have not previously undergone curative-intent PDAC resection and who have a residual primary pancreatic lesion, the pancreatic primary should be included in the radiotherapy field, with a prescribed dose of 33 Gy in 5 fractions (33 Gy/5f). The priority order for selecting metastatic lesions for irradiation is: liver, lung, lymph nodes, bone, and peritoneal nodules (**Table 1**). To improve consistency across participating centers, SBRT planning and delivery will follow protocol-specified guidance for target selection, dose/fractionation, treatment planning, and organ-at-risk protection. Organ-at-risk dose limitations will be applied with reference to relevant authoritative domestic and international guidelines and consensus recommendations, including the CSCO pancreatic cancer guidelines and the ESTRO ACROP Guidelines for Target Volume Definition in Pancreatic Cancer (18, 19). Final radiotherapy planning will be individualized by the treating radiation oncologist according to lesion location, adjacent organs at risk, and normal tissue tolerance. When target coverage conflicts with organ-at-risk protection, organ-at-risk constraints will take priority.

**Table 1:** Recommended radiation therapy dosages by site.

Site (target lesion)	Preferred dose-fractionation	Acceptable alternatives
Primary pancreatic lesion	6.6 Gy × 5 fractions	30–40 Gy/5 fractions; or 25 Gy/5 fractions
Liver metastasis	50 Gy/5 fractions	35–50 Gy/5 fractions; or 25 Gy/5 fractions
Lung metastasis	50 Gy/5 fractions	35–50 Gy/5 fractions; or 25 Gy/5 fractions
Lymph node metastasis	6.6 Gy × 5 fractions	30–45 Gy/5 fractions; or 25 Gy/5 fractions
Bone metastasis (non–spinal cord related)	35 Gy/5 fractions	30–50 Gy/5 fractions; or 25 Gy/5 fractions
Bone metastasis (spinal cord related)	30 Gy/5 fractions	30–50 Gy/5 fractions; or 25 Gy/5 fractions
Peritoneal metastasis	30 Gy/5 fractions	30–50 Gy/5 fractions; or 25 Gy/5 fractions

Eligible patients will have no more than 10 metastatic lesions in total. When the total number of lesions is >5, not all lesions are required to be included in the radiotherapy target volume; when the total number of lesions is ≤5, target coverage should be achieved as completely as possible. However, if lesions are large or the intended target lesion is adjacent to critical organs at risk, the radiation oncologist may individualize lesion selection. In such cases, GTV delineation and treatment planning should follow the principle of compromising target coverage to meet organ-at-risk dose constraints; when necessary, lesions located farther from organs at risk may be selected as substitutes for higher-priority lesions.

Patients who complete at least 8 cycles of treatment without progression may receive maintenance therapy with “gemcitabine plus Serplulimab” in the experimental group, while the control group receives gemcitabine monotherapy maintenance. If progression occurs during maintenance, reintroduction of the initial combination regimen is permitted. Upon subsequent progression, patients transition to a second-line regimen (e.g., nal-IRI + 5-FU/LV). Additionally, patients meeting criteria following MDT assessment may undergo curative surgery. Postoperative follow-up will continue to monitor survival outcomes and subsequent antitumor treatment information.

### 2.3 Eligibility and enrollment

This study will enroll patients aged 18–75 years with an ECOG performance status of 0–2, an expected survival of at least 3 months, and pathologically confirmed recurrent or metastatic PDAC not amenable to curative treatment. Eligible patients must be treatment-naïve for recurrent/metastatic disease. Prior neoadjuvant and/or adjuvant therapy is permitted only if disease recurrence occurs more than 6 months after completion of perioperative treatment.

Patients must have at least one measurable lesion according to RECIST v1.1 and at least one lesion considered suitable for radiotherapy. A radiotherapy-suitable lesion is defined as a lesion that can be safely treated with protocol-specified SBRT on the basis of lesion location, size, and anatomical relationship to adjacent critical organs at risk. In particular, the lesion must be technically amenable to target delineation and treatment planning, and must not be so close to high-risk organs, such as the stomach, duodenum, small bowel, or spinal cord, that protocol-specified radiotherapy cannot be delivered safely while respecting organ-at-risk protection. Prior radiotherapy to the intended target lesion is not permitted. Patients with more than 10 metastatic lesions in total, or with lesions deemed unsuitable for safe protocol-specified radiotherapy, will not be eligible. Adequate hematologic, hepatic, renal, and coagulation function is required before enrollment.

Key exclusion criteria include prior systemic therapy for recurrent/metastatic disease, prior immunotherapy, uncontrolled central nervous system metastases, active autoimmune disease requiring systemic treatment, uncontrolled infection, significant cardiovascular or cerebrovascular disease, major bleeding or thrombotic risk, recent immunosuppressive treatment, recent live vaccination, and other conditions judged by investigators to compromise protocol compliance or patient safety. Detailed inclusion and exclusion criteria are provided in **Supplementary Tables 1**, **2**.

Patients are enrolled in the study after signing a written informed consent form and are formally admitted to the clinical trial upon meeting inclusion and exclusion criteria. Qualified researchers will provide patients with comprehensive information regarding the study’s purpose, procedures, potential risks, benefits, and patient rights. The informed consent process will adhere to Institutional Review Board (IRB) guidelines and local regulations to safeguard patient rights and privacy.

### 2.4 Follow-up plan

The screening period typically lasts 28 days. After signing the informed consent form, subjects must complete a comprehensive baseline assessment. This includes collection of demographic data, detailed medical history, and prior antitumor treatment history, along with a full physical examination, ECOG performance status assessment (within 7 days prior to first dosing), electrocardiogram (ECG), and echocardiogram (LVEF) evaluation. Additionally, routine blood and urine tests, biochemistry, coagulation studies, cardiac function markers, thyroid function, and virological screening (HIV/HBV/HCV) are required. Tumor tissue samples (for PD-L1, MSI, etc.) and blood samples meeting specified criteria must be collected. Baseline imaging (CT/MRI) must be completed within 28 days prior to the first dose to identify measurable lesions and establish a baseline for subsequent efficacy assessment.

Subsequently, subjects receive study drug treatment cyclically until disease progression (PD), intolerable toxicity, or withdrawal of informed consent. Prior to each dosing, vital signs, body weight (dose adjustment required for >10% change), ECG, ECOG performance status, and laboratory parameters (complete blood count, biochemistry, coagulation every cycle; thyroid and virology every 2 cycles) must be monitored. Efficacy assessment involves imaging every 6 weeks (± 7 days). If a subject first demonstrates PD per RECIST 1.1 criteria but the investigator determines ongoing clinical benefit and compliance with relevant standards, subjects in the treatment arm may continue therapy after re-signing informed consent, with imaging confirmation required 4–8 weeks later.

Following treatment discontinuation, subjects must complete a final visit and undergo multi-phase follow-up. Safety follow-up includes a visit 30 days after last dose (including laboratory tests and physical examination) and a telephone AE follow-up 90 days after last dose. For subjects discontinuing treatment for reasons other than disease progression, imaging assessments should continue every 6 weeks until progression or initiation of new therapy. Thereafter, subjects enter the survival follow-up period, during which investigators will record survival status and subsequent antitumor treatment via telephone every 12 weeks until death or study completion.

### 2.5 Statistical analysis

All statistical analyses will be prespecified in a statistical analysis plan finalized prior to database lock. Efficacy analyses will be primarily conducted in the intention-to-treat (ITT) population, defined as all randomized participants analyzed according to the assigned treatment arm. A per-protocol set (PPS), consisting of participants without major protocol deviations and who receive protocol-specified treatment as planned, will be used for supportive analyses. Safety analyses will be performed in the safety set (SS), defined as all participants who receive at least one dose of any study treatment, analyzed according to the treatment actually received.

Time-to-event endpoints, including overall survival (OS; primary endpoint), progression-free survival (PFS), time to progression (TTP), and duration of response (DOR), will be summarized using the Kaplan–Meier method. Median event times with two-sided 95% confidence intervals (CIs) will be reported. Between-group comparisons for OS and other time-to-event endpoints will be performed using a log-rank test with a one-sided significance level of 0.05 for the primary endpoint. Treatment effects will be estimated using a Cox proportional hazards model and expressed as hazard ratios (HRs) with two-sided 95% CIs.

Objective response rate (ORR) and disease control rate (DCR) will be summarized as proportions with two-sided 95% CIs (Clopper–Pearson method). Between-group differences for binary endpoints will be assessed using a chi-square test or Fisher’s exact test, as appropriate. In addition to overall response per RECIST version 1.1, lesion-specific response endpoints (irradiated-lesion-specific ORR and non-irradiated-lesion-specific ORR) will be analyzed descriptively and comparatively using the same approach.

Eligible subjects in this study will be randomly assigned to the treatment group and control group in a 1:1 ratio. The sample size calculation was based on the median OS of 8.5 months observed in the GnP regimen of the MPACT study (5). We anticipate that the combination therapy in the treatment group will extend patient OS to 13 months. The one-sided significance level is set at 0.05, with a test power of 80%. Each treatment group requires 90 patients, totaling 180 patients. Accounting for approximately 10% loss to follow-up, 198 patients need to be enrolled.

### 2.6 Efficacy and toxicity assessment procedures

The efficacy assessment in this study primarily follows RECIST v1.1 and iRECIST criteria, evaluated jointly by the study center and an independent imaging committee. Safety evaluation encompasses laboratory tests, electrocardiograms, ECOG performance status, and vital signs monitoring. Adverse events (AEs) are defined as any adverse medical events occurring after drug administration, including laboratory abnormalities and exacerbation of underlying conditions. Adverse events leading to death, life-threatening conditions, hospitalization, or disability were classified as serious adverse events (SAEs). Investigators graded the severity of AEs from 1 to 5 according to the NCI CTCAE criteria and assessed causality with the study drug based on temporal relationship and known toxicity. Causality classified as “definite,” “probable,” or “unclassifiable” was considered drug-related adverse reactions.

### 2.7 Dose modifications

Adjustments to chemotherapy drugs (gemcitabine and albumin-bound paclitaxel) follow the principle of “delay, dose reduction, or discontinuation.” Prior to administration in each cycle, subjects must meet the following criteria: neutrophil count ≥1.5×10^9^/L and platelet count ≥80×10^9^/L. In cases of intolerable toxicity, dose delay or reduction is permitted, but each chemotherapy agent may only be reduced twice (dose escalation steps: 1000/125 mg/m² → 800/100 → 600/75). A third dose reduction necessitates discontinuation of chemotherapy. Permanent discontinuation is required for Grade 4 non-hematologic toxicity or recurrence of specific Grade 3 toxicity. Additionally, chemotherapy may be paused for a maximum of 42 consecutive days (6 weeks). Exceeding this duration necessitates discontinuation, and reduced doses may not be reinstated after toxicity resolution.

The core principle for adjusting the dose of Serplulimab is to permit dose interruption but strictly prohibit dose reduction. Throughout the study, the dose of Serplulimab remains unchanged. If a subject experiences an immune-related adverse event (irAE), the investigator may suspend treatment until toxicity resolves to Grade 0–1 or baseline. For suspected pulmonary or hepatic toxicity after SBRT plus Serplulimab, differential diagnosis should consider timing, field distribution, laboratory pattern, and multidisciplinary review. Radiation injury is typically field-conforming; immune-related toxicity more often appears bilateral, multifocal, or non-field-conforming (20). Radiation-induced liver injury may show alkaline phosphatase elevation, whereas immune-related hepatitis is usually transaminase-predominant. Other causes should be excluded when clinically indicated in practice (21). Typically, the maximum allowable consecutive suspension period for Serplulimab is 12 weeks. Treatment must be discontinued if suspension exceeds 12 weeks (except for delays due to steroid tapering or force majeure). Permanent discontinuation of slurelimab is required for life-threatening Grade 4 toxicity, severe Grade 3 irAE (e.g., pneumonia, colitis, nephrotoxicity that does not improve with treatment), or severe Grade 3–4 infusion reactions. If chemotherapy cycles are delayed, slurelimab administration may be postponed at the investigator’s discretion to maintain synchronization.

## 3 Discussion

PDAC exhibits a highly aggressive biological behavior, an insidious disease progression, and widespread resistance to conventional therapeutic approaches, collectively contributing to its extremely poor prognosis. Currently, the standard first-line treatments for metastatic PDAC are the GnP and FOLFIRINOX regimens (22). In the present triplet strategy, GnP was selected as the chemotherapy backbone because it is widely used in routine practice and has a clinically manageable safety profile in real-world and Chinese settings, while its day 1/day 8 schedule is also easier to coordinate with SBRT and close toxicity monitoring during multimodality treatment (23). However, for most patients, single-agent chemotherapy regimens fail to address the immune evasion and drug resistance issues arising from the complex tumor microenvironment of PDAC. The WGOG-PAN 006/ICSBR-2 study introduced serplulimab, a novel anti-PD-1 monoclonal antibody, combined with SBRT, aims to overcome immune resistance barriers in PDAC through a synergistic model combining chemotherapy-induced tumor shrinkage with radiotherapy-enhanced immunotherapy. Its core mechanism lies in transforming “cold tumors” into “hot tumors” via SBRT-induced immunogenic cell death, thereby creating conditions conducive to immune checkpoint inhibitor efficacy (24).

Immune checkpoint inhibitors have achieved revolutionary success in tumors such as melanoma, but their efficacy as monotherapy in PDAC is virtually nonexistent except in the rare MSI-H/dMMR population (25). This widespread resistance is not only related to the microenvironment but also critically influenced by the affinity and mechanism of action of the antibodies themselves. Serplulimab, as a recombinant humanized monoclonal anti-PD-1 antibody, possesses multiple design advantages that differentiate it from others. Research has demonstrated that serplulimab possesses the ability to induce PD-1 receptor endocytosis. Furthermore, the complex formed when it binds to PD-1 exhibits weaker cis interference with CD28 co-stimulatory signaling. Under physiological conditions, PD-1 binding to its ligand recruits SHP2 phosphatase, leading to CD28 dephosphorylation and subsequent inhibition of T cell activation. Serplulimab mitigates this inhibitory effect by optimizing its binding epitope, enabling T cells to maintain stronger antitumor activity (26). Additionally, Serplulimab has demonstrated significant survival benefits in highly challenging tumor types such as small cell lung cancer (ASTRUM-005) and esophageal squamous cell carcinoma (ASTRUM-007), providing important insights for PDAC treatment (27, 28). In a prior Phase II study, the combination of Serplulimab with GnP and SBRT achieved an ORR of 74.47% and a DCR of 100%, substantially surpassing historical data from single-agent chemotherapy. This further confirms Serplulimab’s potent synergistic potential in combination therapy (17).

The dense interstitial fibrosis of PDAC, coupled with hypoxia and an acidic environment resulting from sparse vascular distribution, forms a physical and biochemical barrier that impedes the penetration of chemotherapeutic drugs and limits the recruitment of effector T cells to the tumor core (24). As a high-dose, precision radiotherapy modality, SBRT has emerged as a game-changer in research. The biological effects of SBRT extend beyond direct tumor cell killing. Under precisely targeted high-dose irradiation, tumor cells undergo immunogenic cell death, leading to the ectopic expression of calreticulin on the cell membrane surface and the release of ATP and HMGB1 protein. These molecules, termed damage-associated molecular patterns, effectively activate dendritic cells and promote the presentation of tumor-associated antigens. This process effectively transforms PDAC lesions, which were previously quiescent or immunologically constrained, into an endogenous “*in situ* vaccine (29).”Furthermore, SBRT enhances interferon production via the cGAS-STING signaling pathway, upregulating MHC-I molecule expression on tumor cell surfaces to improve CTL recognition and killing of tumor cells (30). Crucially, radiotherapy reshapes the cellular composition of the microenvironment. Research indicates that optimally dosed radiation induces tumor-associated macrophages to switch from the tumor-promoting M2 phenotype to the anti-tumor M1 phenotype. In parallel, RT-based immune priming is expected to enhance the infiltration and functional reinvigoration of tumor-reactive CD8+ T cells, including partial reversal of exhausted states in the context of PD-1 blockade (31, 32). This multimodality approach may also help counteract lactate-rich metabolic immunosuppression within PDAC, thereby relieving suppression of effector T-cell activity in the acidic stromal niche (33). Through this mechanism, SBRT disrupts the immune suppression balance characteristic of PDAC, paving the way for Serplulimab-driven immune activation (34).

Although the triple-combination regimen shows tremendous potential in efficacy, its safety profile remains a primary concern for clinical experts. The complex safety landscape encompasses bone marrow suppression and neurotoxicity inherent to chemotherapy regimens, immune-related adverse events potentially arising from combination immunotherapy, and the risk of potential damage to hollow organs from SBRT (6). This study incorporated multiple protective measures in its design. First, at the technical execution level of SBRT, the prescribed dose for primary lesions located in the pancreatic head was strictly limited to 33 Gy/5f to ensure that the radiation dose to OARs such as the duodenum remained within the safety threshold (D0.5cc < 33 Gy). For metastatic lesions in organs with higher radiation tolerance, such as the liver and lungs, the dose could be escalated to 50 Gy to maximize local control and antigen release. Second, regarding dose adjustment, the study established detailed principles for stepwise dose reduction. Notably, the adjustment principle for Serplulimab is “permit suspension but not dose reduction,” aiming to ensure effective concentrations for immune checkpoint blockade and thereby maintain long-term T-cell activation. Based on current preliminary safety data, the incidence of Grade 3/4 treatment-related adverse events (TRAEs) for the SBRT triple-agent regimen is approximately 52.8%, primarily concentrated in hematologic toxicity (17). This represents no significant increase compared to historical data for the GnP monotherapy regimen (e.g., 40-50% in the MPACT study). Immune-related serious adverse events (such as Grade 3 or higher rash, myocarditis, or pneumonia) occurred at a low rate (<10%) and were effectively controlled through timely corticosteroid intervention. This safety profile provides a solid foundation for scaling up to larger patient populations.

## 4 Conclusion

This randomized, open-label, multicenter Phase III trial (WGOG-PAN 006/ICSBR-2) will prospectively evaluate whether adding serplulimab and metastasis-directed SBRT to standard gemcitabine plus nab-paclitaxel can improve overall survival for treatment-naive patients with recurrent or metastatic PDAC. By integrating cytotoxic debulking, SBRT-driven immune priming, and PD-1 blockade, the study is designed to test a biologically rational “radio-immuno-chemotherapy” strategy intended to overcome the immunologically cold and stromal-restricted tumor microenvironment that limits current outcomes. If the primary endpoint is met, the results could support a practice-changing first-line approach for a broad PDAC population and provide a mechanistic framework for optimizing radiotherapy–immunotherapy combinations in this disease.

## Data Availability

The original contributions presented in the study are included in the article/**Supplementary Material**. Further inquiries can be directed to the corresponding authors.
