# Supplementary material for: Serplulimab combined with gemcitabine, nab-paclitaxel, and stereotactic body radiotherapy versus gemcitabine and nab-paclitaxel as first-line treatment for recurrent or metastatic pancreatic ductal adenocarcinoma: a randomized, open-label, multicenter, phase III clinical trial (WGOG-PAN 006/ICSBR-2)
Source: Front Immunol. 2026 May 13;17:1817221. doi: 10.3389/fimmu.2026.1817221 (PMC13212061; doi:10.3389/fimmu.2026.1817221)
Supplement: Supplementary file 1 [file Table1.docx]

1. Detailed Inclusion Criteria

| **Inclusion Criteria** | **Details** |
| --- | --- |
| 1 | Patients with pathologically confirmed metastatic pancreatic cancer, unresectable; diagnosis determined by pathology plus imaging (CT or MRI). |
| 2 | No prior systemic antitumor therapy (including chemotherapy, radiotherapy, or other investigational therapy); OR prior curative-intent resection with standard neoadjuvant/adjuvant chemotherapy, with recurrence/progression >6 months after the last adjuvant chemotherapy. |
| 3 | Age 18–75 years, any sex. |
| 4 | ECOG performance status 0–2. |
| 5 | At least one measurable lesion: spiral CT with longest diameter ≥10 mm, or lymph node short axis ≥15 mm; on conventional CT or physical exam, maximum diameter ≥20 mm. |
| 6 | Excluding the primary lesion: ≤10 metastatic lesions in total; largest metastatic lesion ≤10 cm; and ≥1 lesion suitable for radiotherapy based on imaging assessment. |
| 7 | Adequate major organ function (as defined below). |
| 7a | Bone marrow function (no blood transfusion within 14 days prior to screening): WBC ≥3.0×10^9/L; ANC ≥1.5×10^9/L; PLT ≥80×10^9/L; Hb ≥90 g/L. |
| 7b | Hepatic function: ALT and AST ≤3×ULN; TBIL ≤1.5×ULN. If liver metastases are present: ALT and AST ≤5×ULN and TBIL ≤2×ULN are allowed. Child–Pugh score ≤7. |
| 7c | Renal function: serum creatinine ≤1.5×ULN; proteinuria ≤2+ or ≤2 g/24 h; GFR ≥60 mL/min/1.73 m² (calculated by the Cockcroft–Gault formula). |
| 7d | Coagulation function: PT, APTT, and INR ≤1.5×ULN. Patients receiving a fixed anticoagulation regimen for ≥30 days prior to study drug may have PT or INR >1.5×ULN if deemed appropriate by the investigator, with sufficient justification provided. |
| 7e | Electrolytes: serum sodium, potassium, calcium, and magnesium abnormalities ≤ Grade 1 per NCI-CTCAE v5.0. |
| 7f | ECG: QTc interval ≤480 ms. |
| 8 | Expected survival ≥3 months. |
| 9 | Women of childbearing potential and men with partners of childbearing potential must use ≥1 medically accepted contraceptive method during study treatment and for ≥6 months after the last dose of chemotherapy or anti–PD-1 antibody. |
| 10 | Able to understand the protocol, comply with study procedures, and provide written informed consent before any study-related procedures. |

Notes:

(1) “Treatment-naive for recurrent/metastatic disease” means that no prior systemic antitumor therapy is permitted for recurrent or metastatic PDAC. Prior neoadjuvant and/or adjuvant therapy is permitted only if recurrence or progression occurs more than 6 months after completion of perioperative treatment.

(2) “A lesion suitable for radiotherapy” refers to a lesion that can be safely treated with protocol-specified SBRT according to lesion location, lesion size, and its anatomical relationship to adjacent critical organs at risk. In particular, the lesion must be technically amenable to target delineation and treatment planning, and must not be so close to high-risk organs that protocol-specified radiotherapy cannot be delivered safely while respecting organ-at-risk protection. Relevant organ-at-risk assessment will be performed with reference to authoritative domestic and international guidelines and consensus recommendations for pancreatic radiotherapy, including consideration of structures such as the stomach, duodenum, small bowel, spinal cord, kidneys, and liver.

(3) Prior radiotherapy to the intended target lesion is not permitted.

(4) Patients must provide written informed consent before any study-related procedures.
