# Supplementary material for: Serplulimab combined with gemcitabine, nab-paclitaxel, and stereotactic body radiotherapy versus gemcitabine and nab-paclitaxel as first-line treatment for recurrent or metastatic pancreatic ductal adenocarcinoma: a randomized, open-label, multicenter, phase III clinical trial (WGOG-PAN 006/ICSBR-2)
Source: Front Immunol. 2026 May 13;17:1817221. doi: 10.3389/fimmu.2026.1817221 (PMC13212061; doi:10.3389/fimmu.2026.1817221)
Supplement: Supplementary file 2 [file Table2.docx]

1. Detailed Exclusion Criteria

| **Exclusion Criteria** | **Details** |
| --- | --- |
| 1 | Known allergy to any investigational product. |
| 2 | Known or suspected central nervous system (CNS) metastases, i.e., signs/symptoms suggestive of CNS involvement, unless CNS metastases are ruled out by CT or MRI. |
| 3 | History of other malignancies within 5 years before first investigational product administration (except adequately treated basal cell carcinoma of skin or carcinoma in situ of cervix). |
| 4 | Prior treatment with immunotherapy agents, including anti–PD-1, anti–PD-L1, anti–CTLA-4, etc. |
| 5 | Requirement for concomitant antitumor therapies outside the study regimen during the trial, including but not limited to chemotherapy, targeted therapy, hormonal therapy, immunotherapy, radiotherapy, or antitumor traditional Chinese medicine. |
| 6 | Diagnosis of immunodeficiency or receipt of other forms of immunosuppressive therapy within 7 days prior to first dose. |
| 7 | Systemic treatment with corticosteroids (>10 mg/day prednisone equivalent) or other immunosuppressants within 14 days prior to first dose. Inhaled or topical steroids are allowed; adrenal corticosteroid replacement at ≤10 mg/day prednisone equivalent is allowed in the absence of active autoimmune disease. |
| 8 | Prior anticancer vaccination, or receipt of a live vaccine within 4 weeks prior to first dose. |
| 9 | Major surgery within 28 days prior to first dose (defined as surgery requiring ≥3 weeks of recovery before study treatment can be initiated). Tumor biopsy or lymph node excisional/incisional biopsy is allowed. |
| 10 | Concomitant medications required during the study that may affect study drug metabolism. |
| 11 | Uncontrollable third-space effusions (e.g., pleural effusion, pericardial effusion, ascites). |
| 12 | Uncontrolled cardiovascular or cerebrovascular conditions/symptoms, including but not limited to: NYHA class > II heart failure, unstable angina, myocardial infarction or cerebral infarction within 6 months, clinically significant supraventricular/ventricular arrhythmias not adequately controlled with intervention. |
| 13 | Uncontrolled hypertension (post-treatment SBP >160 mmHg and/or DBP >100 mmHg). |
| 14 | History of severe bleeding or thromboembolic events within 6 months, e.g., cerebrovascular accident (including TIA), pulmonary embolism, spontaneous massive tumor bleeding, etc. |
| 15 | Severe infection within 4 weeks prior to first dose (CTCAE grade >2), such as severe pneumonia requiring hospitalization, bacteremia, infectious complications; baseline chest imaging showing active pulmonary inflammation with clinically relevant symptoms/signs; infection symptoms/signs within 2 weeks prior to first dose or requiring oral/IV antibiotics (excluding prophylactic antibiotics). |
| 16 | Known active autoimmune disease (e.g., interstitial pneumonitis, colitis, hepatitis, hypophysitis, vasculitis, nephritis, hyperthyroidism, hypothyroidism, etc.). Exceptions: vitiligo; resolved childhood asthma/allergy without adult intervention; autoimmune-mediated hypothyroidism on stable thyroid replacement; type 1 diabetes on stable insulin. Subjects in a stable condition not requiring systemic immunosuppression (including systemic corticosteroids) may be eligible. |
| 17 | History of immunodeficiency, including HIV antibody positive, other acquired or congenital immunodeficiency, history of organ transplantation, or allogeneic bone marrow transplantation. |
| 18 | Active tuberculosis detected by history or CT; active TB within 1 year prior to enrollment; or active TB >1 year ago without standard treatment. |
| 19 | HBsAg positive and/or HBcAb positive with HBV-DNA ≥500 IU/mL (or ≥2500 copies/mL) at enrollment. If above threshold, subject must receive antiviral therapy first and decrease to normal for ≥2 weeks, and continue antiviral therapy throughout the study. Subjects who require or are receiving antiviral therapy must continue throughout the study even if HBV-DNA meets eligibility. HCV: anti-HCV positive with HCV-RNA positive. |
| 20 | Concomitant medications judged by the investigator to affect metabolism of the investigational product, e.g., strong CYP3A4 inhibitors/inducers, or drugs mainly metabolized by CYP3A4, 2C8, 2C9, 2C19, or 2D6 with a narrow therapeutic index. |
| 21 | Pregnant or breastfeeding women, or subjects of childbearing potential unwilling to use contraception during the trial and for 3 months after the last dose. |
| 22 | Any other condition judged by the investigator to potentially lead to premature discontinuation, such as other serious diseases (including psychiatric disorders) requiring concomitant treatment, markedly abnormal laboratory values, or family/social factors that may compromise subject safety or data collection; deemed unsuitable for enrollment by the investigator. |

Notes:

(1) Patients will be excluded if the intended target lesion has received prior radiotherapy, or if prior irradiation would preclude safe delivery of protocol-specified SBRT.

(2) Patients will also be excluded if, in the judgment of the treating radiation oncologist, protocol-specified SBRT cannot be delivered safely because of lesion size, lesion location, or an unfavorable anatomical relationship to adjacent critical organs at risk.

(3) The final determination of radiotherapy feasibility and safety will be made comprehensively according to protocol-specified planning principles and organ-at-risk protection requirements.
